# Endocrinopathies in Survivors of Childhood Neoplasia

**DOI:** 10.3389/fped.2014.00101

**Published:** 2014-09-23

**Authors:** Nicole Barnes, Wassim Chemaitilly

**Affiliations:** ^1^Division of Pediatric Endocrinology, Department of Pediatric Medicine, St. Jude Children’s Research Hospital, Memphis, TN, USA; ^2^Epidemiology and Cancer Control, St. Jude Children’s Research Hospital, Memphis, TN, USA

**Keywords:** endocrinology, growth, puberty, gonads, childhood cancer survivors, thyroid disorders

## Abstract

Advancements in cancer treatments have increased the number of survivors of childhood cancers. Endocrinopathies are common complications following cancer therapy and may occur decades later. The objective of the current review is to address the main endocrine abnormalities detected in childhood cancer survivors including disorders of the hypothalamic-pituitary axis, thyroid, puberty, gonads, bone, body composition, and glucose metabolism.

## Introduction

**Figure 1:**
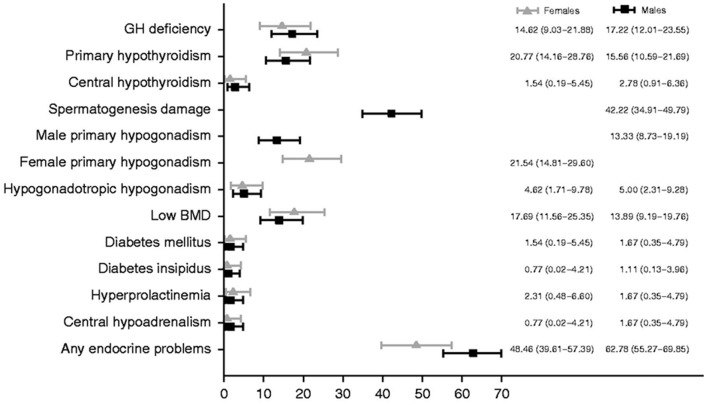
**Prevalence of endocrine disorders at the last follow-up visit by gender**. Reproduced with permission from Ref. (4) ©2013 European Society of Endocrinology.

Approximately 1 in 285 children will be diagnosed with cancer before the age 20 years, and 1 in 530 young adults between the ages of 20 and 39 years is a childhood cancer survivor (CCS) (1). Endocrine complications are among the most common sequelae observed in CCS, and they frequently occur as cancer therapy-related late – effects appearing years, even decades, after the exposure to chemotherapy and/or radiotherapy. The prevalence of an endocrine disorder in 1423 at risk adult CCS was reported to be 62% (95% CI 59.5–64.6) (2). The 60-year cumulative risk of having an endocrinopathy in an individual diagnosed with cancer between the ages of 5 and 9 years was 43% in a large cohort of Northern European CCS (3). The occurrence of endocrine disorders documented in an Italian Transition Unit for adult CCS was 48.46 and 62.78% in females and males, respectively (Figure 1) (4). Treatment exposures placing individuals at risk of endocrinopathies have traditionally included alkylating agent based chemotherapy and radiotherapy. More recently, selective mitogen-activated kinase inhibitors and immune system modulators have been shown to also be associated with endocrine dysfunction. The long-term consequences of the use of these novel therapies, some of which are prescribed in maintenance regimens, remain to be fully elucidated (5–7). Healthcare providers should be equipped to diagnose and manage acute and long-term endocrine complications that may arise in maturing CCS. This review will address the risk of endocrine disorders associated with the treatment of pediatric cancer and brain tumors. The data summarized in this review are based on a systematic search of the medical literature using MEDLINE/Pubmed (from 1970 to May 2014) using keywords relevant to this topic. Additional searches were conducted within the reference lists of relevant articles.

## Disorders of the Hypothalamus and Pituitary

Tumor development and/or surgical resections close to the hypothalamus and/or pituitary may induce direct anatomical damage to these structures and result in multiple hypothalamic/pituitary dysfunctions (Table 1). Disorders of the hypothalamus/pituitary are also common following their exposure to direct or scatter radiotherapy. More recently, Ipilimumab, an immune system modulator, was shown to potentially cause auto-immune hypophysitis with ensuing anterior panhypopituitarism (7). Pituitary dysfunction was the most frequent endocrine complication in a Northern European cohort comparing 31,723 CCS and 211,261 controls. In this study, the standard hospitalization rate ratio of hypopituitarism was 88.0 (95% CI 72.1–107.5) in CCS when compared to matched controls from the local general population (3).

**Table 1:** **Central endocrinopathies**.

Function	Complication	Therapy-related risks	Relationship to time, dose to gland, or organ when applicable	Evaluation/labs	Intervention
Linear growth	GH deficiency	Surgery	Damage to the pituitary by tumor expansion and/or surgery	Bone age IGF1, IGF-BP3	GH replacement
		Radiotherapy to hypothalamus/pituitary	Doses ≥18 Gy (highest risk ≥30 Gy)	GH stimulation test	

Puberty	Central precocious puberty	Radiotherapy to hypothalamus/pituitary	Doses ≥18 Gy,	Bone age	GnRH agonist
			Girls <5 years old at exposure have a higher risk	Baseline AM LH, FSH, estradiol (girls), or testosterone (boys)	
				Leuprolide stimulation test	
	LH/FSH deficiency^a^	Surgery	Damage to the pituitary by tumor expansion or growth	Bone age Baseline AM LH, FSH, estradiol (girls), or testosterone (boys)	Induction of puberty/sex hormone replacement therapy
		Radiotherapy to hypothalamus/pituitary	Doses ≥30 Gy		
			Partial deficit ≥20 Gy	

Pituitary, other	ACTH insufficiency^a^	Surgery	Damage to the pituitary by tumor expansion and/or surgery	8 a.m. cortisol and ACTH	Hydrocortisone and stress dose teaching
		Irradiation to hypothalamus or pituitary	Doses ≥30 Gy	Low dose ACTH stimulation test if AM cortisol is abnormal	
		Systemic glucocorticoids	Deficiency depends on the doses used and duration of exposure	
	TSH deficiency^a^	Surgery	Damage to the pituitary by tumor expansion and/or surgery	Free T4	Levothyroxine
		Radiotherapy to hypothalamus/pituitary	Doses ≥30 Gy	
	Central diabetes insipidus	Surgery	Damage to the pituitary by tumor expansion and/or surgery	Plasma electrolytes, serum, and urinary osmolalities. Water deprivation test in equivocal situations	Desmopressin Fluid management

*GH, growth hormone; IGF-1, insulin-like growth factor-1; IGF-BP3, insulin-like growth factor binding protein 3; GnRH, gonadotropin releasing hormone; ACTH, corticotropin; TSH, thyroid stimulating hormone; AM, morning sample; LH, luteinizing hormone; FSH, follicle-stimulating hormone*.

*^a^Also described in the context of ipilimumab-induced anterior hypophysitis*.

### Growth hormone deficiency and poor linear growth

Growth failure and short stature are among the most common sequelae of childhood cancer therapy (8). Several etiologies may contribute to growth failure in CCS including growth hormone deficiency (GHD), exposures to spinal and total-body irradiation (TBI), pubertal disorders, chemotherapy treatments including glucocorticoids, hypothyroidism, suboptimal nutrition, and renal disease (9–12).

**Figure 2:**
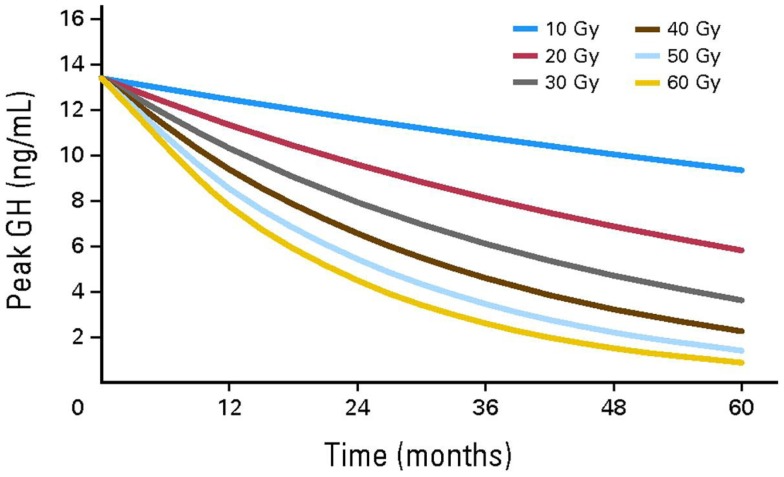
**Growth hormone secretion after hypothalamic/pituitary exposures to radiotherapy**. Reproduced with permission from Ref. (13) ©2011 by American Society of Clinical Oncology.

In CCS, GHD is frequently attributed to cranial radiotherapy doses of 12–64 Gy to the hypothalamus/pituitary (4). Radiation has a dose and time dependent effect on GH secretion. Merchant et al. demonstrated that GHD was likely to develop within 36 months of exposure to hypothalamic/pituitary radiotherapy in individuals receiving doses ≥20 Gy (13) (Figure 2). In comparison to radiotherapy, the impact of chemotherapy alone on GHD secretion is more controversial and less common (14–17). Imatinib, a tyrosine kinase inhibitor (TKI), has been associated with growth deceleration and with failure of provocative GH stimulation testing (18, 19). Imatinib is presumed to inhibit bone growth by impeding the kinase mediated release of GH (5).

Growth hormone deficiency should be investigated in skeletally immature CCS when linear growth velocity decelerates over a 6-month period. The effect of GHD on growth may be masked by precocious puberty and by hyperinsulinemia in the context of rapid weight gain (“growth without growth hormone”) with seemingly normal linear growth driven by sex steroids and insulin respectively in affected individuals (20, 21). CCS exposed to spinal radiotherapy are at risk of having skeletal disproportions; this should ideally be investigated by measuring the sitting height (12). Biochemical evaluation for GHD requires dynamic testing, which despite limitations related to poor reproducibility in the general population, remains acceptable for the assessment of GH secretion in CCS (22). In the general population, the diagnosis of GHD typically requires failing dynamic tests using two different secretagogues; however, in CCS exposed to cranial radiotherapy and individuals with a history of a brain tumor close to the hypothalamus/pituitary, failing one test was considered sufficient in the consensus guidelines published by the Growth Hormone Research Society (23). Secretagogues used in dynamic testing include insulin, arginine, levodopa, clonidine, and glucagon. GH releasing hormone (GHRH) should not be used for the assessment of GH secretion in this population given the primarily hypothalamic location of radiation-induced damage (24). Plasma levels of IGF-1 and IGFBP3, although commonly practiced, are not reliable screening tools in CCS exposed to cranial radiotherapy and are associated with high rates of false-negatives (25).

Treatment with recombinant GH (rGH) replacement therapy is typically not initiated until 12 months after successfully completing cancer or brain tumor treatments. The mitogenic potential of GH stimulating tumor growth is a safety concern in CCS (26). Studies suggest that rGH in patients with brain tumors are not associated with primary disease recurrence (27–29). However, there may be an increase in the development of second neoplasm in CCS treated with GH (30, 31). Ergun-Longmire et al. reported a relative risk of 2.15 (95% CI, 1.3–3.5; *p* < 0.002) of developing a second neoplasm in CCS treated with rGH when compared to controls and the most commonly identified neoplasms were meningiomas (30). Nevertheless, using the same multi-center cohort of CCS and reporting specifically on the risk of subsequent central nervous system neoplasms after a longer period of follow-up, Patterson et al. recently reported an adjusted rate ratio of meningioma and gliomas in GH treated survivors of CNS tumors when compared to CNS tumor survivors who were not treated with GH of 1.0 (95% CI 0.6–1.8, *p* = 0.94), thus indicating negligible differences between the two groups in regards to this particular risk (32).

The benefits of rGH extend beyond linear growth and are highlighted in adult GHD studies. Some of the advantages include improvements in bone mineral density (BMD), cardiovascular function, reduction in metabolic syndrome, and sustained improvements in quality of life (33). The benefits and risk of rGH have to be carefully weighed in children and adult survivors. Ongoing studies are needed to investigate and characterize the risk of developing second neoplasms as well as the proposed advancements in the physiological and psycho-social well-being of rGH in CCS.

### Disorders of luteinizing hormone and follicle-stimulating hormone

#### Central precocious puberty

Central precocious puberty (CPP) is defined by the early activation of the hypothalamic–pituitary–gonadal axis leading to the onset of puberty prior to the ages of 8 and 9-years in girls and boys, respectively (34, 35). The consequences of CPP include the premature closure of growth plates resulting in decreased adult height prospects. Precocious puberty, especially menarche, can generate significant psycho-social adjustment challenges in young children, particularly in those with special needs. While radiotherapy to the hypothalamus/pituitary is the main risk factor associated with CPP in CCS; pubertal development can also be triggered prematurely by tumors located near the hypothalamus or optic pathways independently from radiation exposure. Additional risk factors include hydrocephalus, female sex, exposure to radiotherapy before the age of 5 years, and increased BMI (36, 37).

The mean linear velocity in children with CPP can be 8–10 cm/year (+2 to +4 SDS for chronological age) at diagnosis (38); however, in CCS the linear growth velocity may be normal or poor secondary to concurrent GHD or spinal damage from radiotherapy (39). Clinicians should not rely on the measurement of testicular volume for the diagnosis of puberty in males exposed to gonadotoxic chemotherapy regimens (as those with alkylating agents) and/or testicular irradiation. In these individuals, treatment-related germ cell injury can impair testicular growth without necessarily affecting their ability to produce testosterone and this can be particularly misleading in the context of CPP. Clinicians should be aware of these caveats and have a low threshold to initiate laboratory testing if there is suspicion of early pubertal growth based on other clinical markers such as penile enlargement, scrotal skin thinning, or pubarche.

Biochemical evidence of CPP includes pubertal basal levels of luteinizing hormone (LH) and sex steroids (estradiol or testosterone). Random basal values may be inconclusive secondary to the pulsatile nature of gonadotropins and stimulated values may be necessary to establish the diagnosis. GnRH agonists are used in stimulation testing and a pubertal LH value and a LH to follicle-stimulating hormone (FSH) ratio >1 is consistent with CPP (34, 40). Radiographic evaluation encompasses an assessment of a child’s skeletal maturation (41, 40). In females a pelvic ultrasound demonstrating pubertal sized uterus and ovaries may also be helpful in confirming the diagnosis (42).

Treatment with a GnRH agonist suppresses the secretion of gonadotropins and may be useful in improving final height prospects by delaying skeletal maturation and allowing a longer time for linear growth (43). This treatment may also act synergistically with rGH and improve the final adult height of GH-deficient CCS who also have CPP (43). Determining the best time to discontinue GnRH agonist therapy even in children with idiopathic CPP can be challenging and requires taking into account multiple factors including chronological age, bone age, target height, psycho-social maturation, and parental preferences. This determination is rendered even more challenging by the possible development over time of permanent LH/FSH deficiency in these patients (44). The use of aromatase inhibitors in order to prolong the delay in closure of growth plates in concert with rGH has been utilized by some clinicians to augment the height outcomes of CCS (45). Data remain inconclusive as to whether or not aromatase inhibitors improve adult height and many pediatric endocrinologists consider their use to be experimental.

#### LH/FSH deficiency

The deficiency in LH/FSH, also referred to as hypogonadotropic hypogonadism, can result in delayed or arrested pubertal development during childhood. The post-pubertal male and female with LH/FSH deficiency may present with androgen insufficiency symptoms and secondary amenorrhea, respectively. LH/FSH deficiency can occur after tumor and/or surgery related damage or after doses of radiotherapy to the hypothalamic–pituitary area >30 Gy (36, 46, 47). Female CCS diagnosed after the age of 10 years and who received doses >50 Gy are at high risk for delayed menarche (36). Deficiency in LH/FSH may also occur in the context of ipilimumab-induced auto-immune hypophysitis, as detailed in Section “Corticotropin Deficiency” (7). Replacement is warranted for the development and maintenance of secondary sex characteristic, optimal bone mass accrual, and body composition. Sex steroids also play a pivotal role in the metabolism of lipids and carbohydrates (48).

### Corticotropin deficiency

Corticotropin (ACTH) deficiency, also known as central adrenal insufficiency, can occur in CCS following tumor and/or surgery related damage or after the exposure of the hypothalamus/pituitary to radiotherapy doses ≥30 Gy (49). Hudson et al. identified disorders of the hypothalamic–pituitary–adrenal axis in 13.8% of CCS exposed to cranial radiotherapy (2). In a study of children with embryonal brain tumors treated with radiotherapy, the 4-year cumulative incidence of ACTH deficiency was 38 ± 6% and there was no significant difference in those patients who received irradiation of >42 and <42 Gy (50). The incidence of ACTH deficiency in patients receiving TBI as part of preconditioning of BMT has been reported at 6% (51) ACTH deficiency was documented in 60% (*n* = 50) of patients receiving BMT for malignant and non-malignant reasons. It was more likely to occur in patients who had been transplanted <1 year prior to testing and all the subjects who were diagnosed were asymptomatic (15). The clustering of ACTH deficiency cases within a year of transplant in this study suggests that a large proportion of patients experience transient forms of this condition as a result of their exposure to high-dose glucocorticoids. More recently, ACTH deficiency has been associated with the use of imatinib, a TKI with 48% of individuals diagnosed 3–71 months following the exposure (52). Ipilimumab, an immune system modulator can cause auto-immune hypophysitis with ensuing panhypopituitarism, especially at doses >3 mg/kg with a reported prevalence of 4.9–17% (7, 53, 54). The onset of hypophysitis may not occur until 6 weeks after therapy and affected individuals may require 2–5 years of glucocorticoid therapy (55). Future investigations are needed to determine when and for how long CCS treated with these agents should be tested. If patients are deemed insufficient, the chronicity of the insufficiency should also be studied.

Symptoms of ACTH deficiency include fatigue, weakness, nausea, vomiting, anorexia, and abdominal cramping. When exposed to a severe illness, patients with ACTH deficiency may develop life threatening complications including hypoglycemia related seizures and hypotensive shock. Untreated ACTH deficiency may be associated with decreased free water clearance and hyponatremia; replacement with hydrocortisone in such instances, especially in post-surgical patients, may unmask co-existent central diabetes insipidus. A screening cortisol level, collected at 8 a.m. that is ≥10 mcg/dl is reassuring and against the presence of ACTH deficiency; a value ≤5 mcg/dl at 8 a.m. should in contrast raise high suspicions regarding this diagnosis. Definitive testing includes insulin tolerance, low dose cosyntropin or metyrapone stimulation tests. A peak cortisol value <18 μg/dl following low dose (1 microgram) cosyntropin is the most commonly used diagnostic criterion (56). The treatment of ACTH deficiency relies on replacement therapy with hydrocortisone and patient and family education regarding stress dosing during times of illness.

### TSH deficiency

Thyroid stimulating hormone deficiency, also known as central hypothyroidism, is rarely reported in CCS. It can occur as a result of tumor and/or surgery related damage or after hypothalamic/pituitary exposure to radiotherapy doses ≥30 Gy (44, 50, 57, 58). One CCS study detected TSH deficiency in 6% (*n* = 71) of childhood brain tumor survivors (59). Deficiency in TSH may also occur in the context of ipilimumab-induced auto-immune hypophysitis, as detailed in Section “Corticotropin Deficiency” (7). Hypothyroidism is associated with poor linear growth, delayed bone age, and disturbances in pubertal timing during childhood. It can also cause fatigue, fluid retention, constipation, cold intolerance, proximal muscle weakness, and depression. A serum free T4 (FT4) below normal in conjunction with a low or normal serum TSH is characteristic of TSH deficiency. Levothyroxine is used to treat this form of hypothyroidism with doses are adjusted to maintain FT4 values within mid to high normal ranges (60). In contrast to primary hypothyroidism (PH), serum TSH values are not helpful in the monitoring of this condition, as they are expected to remain low even after inadequate replacement with levothyroxine.

### Hyperprolactinemia

Prolactin production by the pituitary gland is controlled by the hypothalamus, with a predominantly inhibitory tone due to dopaminergic input. Disruptions of hypothalamic–pituitary connections due to tumor growth, surgery, or doses of radiotherapy >30–50 Gy can result in hyperprolactinemia because of the loss of hypothalamic inhibition on prolactin secretion (44, 61, 62). Patients with hyperprolactinemia can present with galactorrhea. Furthermore, elevated prolactin levels may suppress LH and FSH production and cause hypogonadism. Nevertheless, hyperprolactinemia in CCS tends to remain asymptomatic, especially in a patient population where individuals are concurrently at risk of gonadotropin deficiency and primary gonadal failure because of their cancer treatment exposures (62).

### Central diabetes insipidus

Central diabetes insipidus is the clinical manifestation of the deficit in secretion and release of the anti-diuretic hormone (ADH). Hypothalamic neurons are responsible of the production of ADH; the latter is carried by axonal transport to the posterior pituitary from which it is released into the circulation. The deficiency in ADH impairs the affected individual’s ability to concentrate urine with ensuing polyuria, polydipsia, and dehydration when access to free water is compromised. Central diabetes insipidus can be a mode of revelation of childhood brain malignancies such as dysgerminomas or hypophyseal non-Hodgkin’s lymphomas (63, 64). In these instances, it can initially be isolated and as the tumoral infiltration worsens additional pituitary functions become deficient (64). More commonly, however, diabetes insipidus occurs in the context of pan-hypopituitarism due to the presence of a tumor in close proximity to the sellar region or as a consequence of surgical procedures aimed at removing it. Central diabetes insipidus does not occur as a late effect of cranial radiotherapy and is hence rarely discussed in the literature dedicated to CCS (65). Central diabetes insipidus does not seem to be associated with the use of ipilimumab (7). The management of central diabetes insipidus consists of replacement therapy using desmopressin with close monitoring of fluid intake and urine output in order to avoid overtreatment and ensuing hyponatremia and seizures. Significant shifts in replacement needs are noted in the post-operative patient, mandating close monitoring in the inpatient setting until stabilization is achieved (66, 67). This was well characterized following neurosurgical interventions on sellar/supra sellar tumors such as craniophayngioma with the classical description of a triple phase response consisting of a first phase of transient diabetes insipidus lasting for up to 2 days, followed by an anti-diuretic phase of 1–2 weeks before the onset of permanent diabetes insipidus (67). Patients with altered thirst sensation are more easily managed with the determination of a fixed daily fluid requirement in addition to a fixed dose of desmopressin in order to avoid significant fluctuations in their hydration status (66).

## Disorders of the Thyroid

The radiosensitive nature of the thyroid gland predisposes it to dysfunctions including hypothyroidism, hyperthyroidism, nodules, and cancer (Table 2). Hudson et al. identified thyroid disorders in up to 66.4% of CCS exposed to neck radiotherapy (2). Thyroid dysfunction has been attributed to multiple cancer therapies including radiotherapy with direct or scatter exposure of the neck, TKI, 131 I-Metaidobenzylguanidine ([131I] MIBG), retinoid X receptor agonist autoantibodies, angiogenesis inhibitor thalidomide, and interferon-α (68–70).

**Table 2:** **Peripheral endocrinopathies**.

Function	Complication	Therapy-related risks	Relationship to time, dose to gland, or organ when applicable	Evaluation/labs	Intervention
Thyroid	Primary hypothyroidism	Neck irradiation	Risk increases with dose and time after exposure	TSH, FT4	Levothyroxine
		I131 labeled agents	MIBG for neuroblastoma	
	Hyperthyroidism	Neck irradiation	Doses ≥35 Gy	TSH, FT4, T3	Dependent on clinical course
	Auto-immune hypothyroidism	HSCT	Transfer of auto-immunity from donor	TSH, FT4	Levothyroxine
	Thyroid neoplasms	Neck irradiation	Doses 20–29 Gy	Yearly palpation of neck	Per etiology
			Age <10 at exposure	Thyroid US	
			Females at higher risk	US guided FNAB	

Gonadal disorders male	Leydig cell dysfunction	Testicular irradiation	Doses ≥24 Gy	AM LH, FSH, testosterone	Replacement therapy with testosterone
		Alkylating agents	Generally subclinical	
	Germ cell dysfunction	Testicular irradiation	Possible ≥0.15 Gy	Baseline LH, FSH, inhibin B	Sperm banking
			High risk ≥2 Gy	
		Alkylating agents	Cyclophosphamide dose ≥7.5 gram/m^2^^a^	Adults: semen analysis	
			MOPP ≥3 cycles	
			Busulfan ≥600 mg/m^2^^a^	
			Ifosfamide ≥60 g/m^2^^a^	
			Any alkylating agent in combination with radiotherapy to the testes	

Gonadal disorders female	Ovarian failure	Abdominopelvic irradiation	Acute ovarian failure doses ≥20 Gy	Baseline LH, FSH, estradiol	Induction of puberty with estradiol Hormone replacement therapy Mature oocyte cryopreservation
			Premature menopause/infertility at lower doses		
			Higher risk at older age	Pubertal females-AMH	
		Alkylating agents	Higher risk at older age	

Bone health	Osteoporosis	Radiotherapy	TBI	BMD studies	Per etiology
		Glucocorticoids, methotrexate	Associated hormone deficiencies Nutritional/lifestyle causes	25 Hydroxy-Vitamin D levels	
				Sex Steroids	

Metabolic	Obesity overweight Insulin resistance Metabolic syndrome Diabetes mellitus	Surgery	Hypothalamic injury/central obesity	Waist to Hip Ratio	Lifestyle modifications – diet, physical activity
		Radiotherapy	Cranial radiotherapy abdominal radiation TBI	Fasting: glucose, lipids, insulin, HbA1c	Per etiology
				Oral glucose tolerance if fasting test abnormal	

*TBI, total-body irradiation; TSH, thyroid stimulating hormone; AM, morning sample; LH, luteinizing hormone; FSH, follicle-stimulating hormone; FNAB, ultrasound guided fine needle; aspiration biopsy; TKI, tyrosine kinase inhibitors; AMH, anti-Mullerian hormone; BMD, bone mineral density*.

*^a^Cumulative dose; source: long-term follow-up guidelines for survivors of childhood, adolescent, and young adult cancers – Version 3.0-Oct 2008. Children’s Oncology Group – www.survivorshipguidelines.org*.

### Primary hypothyroidism

Primary hypothyroidism is the most common thyroid abnormality in CCS. In a study by Armstrong et al., the relative risk of PH in CCS was 17.1 when compared to sibling controls (70). The study also documented that up to 50% Hodgkin lymphoma patients receiving >45 Gy developed hypothyroidism within 5-years (70). Female sex and older age at diagnosis were also associated with an increased incidence of hypothyroidism. The risk of PH has primarily been attributed to direct or scatter radiation of the neck including cranio-spinal radiotherapy as well as TBI for cytoreduction before HSCT (50, 58, 59, 71). Subclinical or compensated PH is more commonly diagnosed than overt PH in the context of low dose radiotherapy and HSCT, with some patients experiencing spontaneous recovery (58). Chemotherapy alone has not been traditionally associated with PH. However, TKIs such as sorafenib, sunitinib, and imatinib have been noted to cause thyroid dysfunction (72–74). Hypothyroidism during treatment with sunitinib occurred in 7–85% of patients (72, 73). The pathophysiology of TKI causing PH remains elusive; it may be secondary to destruction of the thyroid gland, impairments of thyroid hormone transport or metabolism, or reduced TSH clearance (72). PH was documented in 13 out 16 CCS of neuroblastoma treated with [131I] MIBG in a long-term follow-up study over a period of 15.5 years (11.2–20.2) (69).

Extended surveillance for thyroid dysfunction is crucial as hypothyroidism in CCS exposed to radiotherapy and radio-labeled agents may occur decades later. The clinical presentation of PH is similar to central hypothyroidism in CCS; however, biochemically they differ. The labs in PH include an elevated plasma TSH level with normal or low free T4; both values are used in monitoring replacement using levothyroxine at substitutive doses.

### Hyperthyroidism

Hyperthyroidism was diagnosed in up to 5% of survivors in a report by Armstrong et al., a rate that was 8 times greater than in sibling controls (70). Overt hyperthyroidism albeit rare has been reported in CCS after hematopoietic stem-cell transplant (HSCT) (75–77). The occurrence of hyperthyroidism may be more common shortly after HSCT with Jung et al. reporting a prevalence of 4.5% in the first 3 months following transplant (77). Lower prevalence values (0.7–2%) have been reported in studies incorporating long-term follow in the pediatric HSCT population (75, 76).

### Autoimmune induced thyroid disease

Positive thyroid autoantibodies have been reported in cases of hypothyroidism and hyperthyroidism in CCS of allogeneic HSCT (78, 79). It has been presumed to be related to the transfer of auto-immunity from the stem-cell donor to the HSCT recipient. However, the presence of thyroid antibodies does not necessarily lead to progression to hypothyroidism in the context of HSCT (80). Auto-immune thyroiditis may exacerbate the thyroid toxicity of certain, but not all, TKI (72, 81). Autoimmune and non-autoimmune thyroiditis are also well established toxicities of interferon-α, a human recombinant cytokine used in the treatment of some solid tumors and hematologic malignancies (82). Newer anticancer agents such as the monoclonal antibodies have also been associated with rare cases of auto-immune hypothyroidism and transient hyperthyroidism. The incidences of ipilimumab-induced auto-immune hypothyroidism in small case reports have been 0–2, 7, and 19% in patients receiving standard doses, high doses, and combination therapy with bevacizumab, respectively (82).

### Thyroid neoplasms

Second thyroid neoplasms may occur later than two decades after the diagnosis of the primary cancer in CCS (83, 84). Armstrong et al. conveyed that 20 years after diagnosis, the risk of having a thyroid nodule in CCS after exposure to neck irradiation was 20%, which is 27 times higher than the sibling population (70). The association between the development of thyroid cancer and direct or scatter radiation of the neck is well known (85). In a large cohort of 12,575 CCS, 111 cases of second primary cancers were pathologically confirmed in patients who had received radiotherapy (86). The most common second primary cancer was papillary thyroid carcinoma and the risk was highest in patients who had received ≤20 Gy, were of female and of a young age (<10 years) at the time of diagnosis of the primary cancer (86). Hodgkin lymphoma is the primary cancer most commonly associated with thyroid cancer. The cumulative incidence of thyroid cancer was 2.3% (95% CI, 1.7–3.1) in this population (83). A recent report by Veiga et al. suggested that alkylating agents in conjunction with <20 Gy of irradiation can increase the incidence of thyroid neoplasm by 2.4 (95% CI, 1.3–4.5; *p* = 0.002) (83). Radiolabeled agents such as 131I MIBG used in the treatment of neuroblastomas have also been associated with the development of papillary thyroid cancer (68).

The currently recommended screening modality for thyroid cancer in CCS at risk is the yearly clinical examination of the neck by an experienced provider. There is significant disagreement regarding the use of thyroid ultrasound for the purposes of screening in the absence of clinical symptoms, because of the high probability of finding abnormal results leading to higher rates of diagnostic procedures and unnecessary anxiety to patients and their families; the yield in identifying malignant nodules with ultrasonography is indeed low, even in survivors of Hodgkin lymphoma (87–90). Kovalchik et al. recently validated an absolute risk prediction model (AUC 0.80, 95% CI), screening for thyroid cancer in CCS. The model was based on sex, age <15 years at primary cancer diagnosis, history of thyroid nodule, radiotherapy to the neck, and exposure to alkylating agents (91). The diagnosis of second primary thyroid cancer in CCS relies on the presence of a positive result on a fine needle aspiration biopsy of a suspected nodule; the treatment approach and prognosis are identical to thyroid cancer cases diagnosed in the general population (87).

## Disorders of the Gonads

### Males

The testes have two functional compartments, a reproductive compartment and an endocrine compartment (Table 2). The reproductive compartment consists of germ cells and the Sertoli cells that support them. The endocrine compartment encompasses the Leydig cells, which are responsible for producing testosterone. The two compartments are affected differently by cancer therapies and understanding this dichotomy in testicular function is imperative in counseling male CCS in regards to their risk of gonadal dysfunction.

#### Leydig cell dysfunction

Hypogonadism after chemotherapy exposure alone is rare (92, 93). However, high doses of alkylating agents, such as cyclophosphamide can cause low testosterone (94, 95). There are also case reports of broad acting kinase inhibitors causing low testosterone and gynecomastia (96). Leydig cell dysfunction as a result of testicular irradiation is dependent on the age of the exposure and the dose of irradiation. Pre-pubertal males receiving doses >24 Gy are at high risk for hypogonadism, whereas pubertal males are at risk when exposed to >33 Gy (97–99). An elevated LH and a low normal morning testosterone define subclinical hypogonadism, and can be attributed to moderate doses of alkylating agent and low dose of testicular irradiation (<20 Gy); however, subclinical hypogonadism rarely requires exogenous testosterone replacement therapy (99–101). At risk, CCS should be followed closely for signs and biochemical evidence of hypogonadism that warrant replacement therapy during pubertal years and adulthood.

#### Germ cell dysfunction

Germ cells are more sensitive to testicular irradiation and chemotherapy than Leydig cells. Chemotherapy agents associated with germ cell dysfunction include cyclophosphamide, procarbazine, ifosfamide, busulfan, melphalan, and cisplatin (99, 102). Green et al. reported germ cell dysfunction with subsequent azospermia in 38.2% (*n* = 275) males exposed to chemotherapy and/or testicular irradiation (103). Small testicular volume, elevated FSH, and low inhibin B levels are associated with poor fertility prognosis in males; however, these clinical and biochemical findings lack sensitivity and specificity in male CCS. Males exposed to gonadotoxic agents may recover germ cell function and it is recommended that they have semen analyses in order to determine fertility status (103, 104).

Sperm cryopreservation is recommended in cancer patients prior to therapy with gonodotoxic agents. In pre-pubertal males, an approach combining testicular tissue extraction, spermatogonial stem-cell preservation, and later transplantation of decontaminated (non-malignant) cells has been proposed but remains experimental (99, 105, 106).

### Females

The ovaries do not replicate the functional dichotomy (distinct endocrine and reproductive compartments) observed in the testes. The female ovarian follicle is responsible for estrogen production and oocyte maturation, and both functions are simultaneously affected by gonadal failure. The extent of ovarian damage and subsequent residual function do not solely depend on the intensity of the cancer treatments received by the patient. These also depend on the number of viable follicles, or “ovarian reserve,” at the time of the exposure. Patients whose viable follicles were depleted during therapy do not experience a recovery of their ovarian function after the completion of cancer treatments and are diagnosed with acute ovarian insufficiency (107). A subset of individuals, with a less severe but nevertheless significant depletion of their ovarian reserve, will experience a resumption of pubertal development or menstrual cycles in the few years following the completion of cancer therapy only to be diagnosed several years later with premature menopause, which is defined as ovarian failure prior to the age of 40 years (108). Given naturally declining numbers of follicles during a woman’s lifespan, ovarian reserve and, consequently, vulnerability to damage from gonadotoxic agents, are particularly dependent on chronological age at the time of exposure to cancer treatments (107, 108). Cancer treatments associated with ovarian toxicity include abdominopelvic irradiation (API) and chemotherapy agents such as cyclophosphamide, procarbazine, busulfan, melphalan, and thiotepa (107–110). Chemotherapy is less toxic to the ovaries of pre-pubertal females in comparison to pubertal and adult females (107, 111–113). The ovarian toxicity of API is age and dose dependent, irradiation doses >20 Gy in female, CCS >13 years old, and TBI in CCS > 10-years is correlated with ovarian insufficiency (107, 114, 115). A study of an AML cohort treated with chemotherapy alone (anthracyclines and cytarabine) demonstrated that menarche occurred at the mean age of 13.1 years and fertility rates were similar to their siblings (92). Even in the absence of exposure to API and despite the occurrence of menarche at a normal age, CCS were nevertheless shown to have a decreased reproductive capacity in comparison to healthy controls in another report (113). The risk of premature menopause was 8 and 0.8% (RR = 13.21, 95% CI = 3.26–53.51; *p* < 0.001) in CCS and siblings, respectively (108). The cumulative incidence of premature menopause was highest among CCS exposed to both alkylating agents and API (108). Evidence of ovarian insufficiency includes elevated gonadotropins, low anti-mullerian hormone (AMH) levels, and reduced mean ovarian volume (113). AMH is an acceptable marker of ovarian follicular reserve in female CCS, and low levels are indicative of declining ovarian function (116–119). Despite declining ovarian reserve in CCS, some survivors have successful pregnancies, with live birth rates of 63–73% (119–121).

Young CCS with ovarian failure may experience poor linear growth and poor bone mineralization. Older hypo-gonadal females can develop menopausal symptoms and are at risk for osteoporosis and cardiovascular disease (122, 123). Sex hormone replacement therapy is warranted in female CCS with ovarian failure. The use of cryopreserved ovarian tissue from pre-pubertal females carries the risk of re-seeding malignant cells and is considered experimental; by contrast, mature oocyte cryopreservation is no longer considered experimental and may represent a viable option for young pubertal females prior to gonadotoxic therapies (124). The availability of this technique, along with better ways of assessing ovarian reserve in females at risk of premature menopause may improve fertility prospects in the CCS population.

## Bone Health Related Complications

Childhood cancer survivors have an increased risk of poor bone health and decreased BMD (Table 2). Contributing factors include the primary cancer (increased osteoclast stimulation in hematological malignancies), treatment exposures, and concurrent hormone deficiencies (125). CCS treated with glucocorticoids and methotrexate or exposed to cranio-spinal radiotherapy, especially receiving >24 Gy of cranial irradiation are susceptible to decreased BMD (125, 126). The association between central nervous system exposures to radiotherapy and low BMD is likely due to radiation related endocrinopathies (deficiencies in GH and/or sex steroids in particular) (126). Lower BMD can be observed prior to cancer therapies because of the effect of the primary illness itself on bone (127). In a recent report on childhood ALL, the 3-year cumulative symptomatic fracture risk was 17.8% (*n* = 399). Fractures were more likely to occur during therapy than during follow up (127). The decline in BMD did not correlate with fracture risk in this study as well as in a report on survivors of osteosarcoma (127, 128). Recent studies have provided further reassurance regarding the continued recovery of BMD after the completion of therapy, a progress that continues, even in older individuals followed through adulthood (126, 129).

Optimizing bone health in CCS includes hormone replacement therapy for those with GHD, hypogonadism, and vitamin D deficiency. It is also recommended that survivors receive adequate nutritional calcium, participate in weight bearing activities, and avoid smoking (125, 128).

## Overweight, Obesity, and Disorders of Glucose Homeostasis

Obesity is a recognized public health challenge with far reaching consequences on overall states of health in the general population (130, 131) (Table 2). The prevalence of obesity and metabolic syndrome in the overall CCS population seems to be comparable to that observed in the general population (2, 130, 132). Hudson et al. demonstrated the prevalence of obesity, hypertension, dyslipidemia, and diabetes was 36.5, 22.6, 50.9, and 5.9%, respectively, in a cohort of CCS followed for 26.3 years after diagnosis (2). Nevertheless, survivors of ALL and brain tumors have significantly higher risks of obesity and overweight (133). Additional risk factors include female sex, doses of cranial radiotherapy >20 Gy, age at exposure <4-years old, and GHD (134, 135). CCS with a history of brain tumor development, radiotherapy, or surgery within the hypothalamus or near the sellar region are at risk of developing a particularly severe form of obesity characterized by hyperphagia and rapid weight gain, and which is sometimes referred to as “central” or “hypothalamic” obesity (136, 137). In these patients with significant hypothalamic injury, increased parasympathetic tone and ensuing hyperinsulinemia (the latter promoting fat storage) have been suggested as possible causes for this phenomenon (136, 137). Treatment approaches for this particular type of obesity have included octreotide and dextroamphetamine (137, 138). In a randomized, double-blind placebo-controlled study of 18 individuals with hypothalamic obesity, octreotide allowed the stabilization of BMI with lower rates of weight gain and lower insulin secretion over a treatment period of 6 months (137). The use of dextroamphetamine in five children with hypothalamic obesity allowed the stabilization of BMI over a period of 24 months (138). The small numbers of patients enrolled in these studies with limited long-term follow-up data to support sustainable efficacy as well as the cost and possible side effects of the medications used in these reports have hindered the wider adoption of such treatment strategies. CCS were also shown to have a higher risk of diabetes mellitus, especially following exposure to TBI, abdominal radiotherapy, and alkylating agents (139, 140). Further research is needed in order to understand the mechanism by which such treatments leave lasting impacts on metabolism and glucose homeostasis as a preamble in optimizing the management of these disorders.

## Summary

Endocrine complications are common in CCS. Healthcare providers need to be aware of the lifelong endocrinopathies associated with treatment exposures. Further research is needed in order to improve risk prediction and develop patient centered screening strategies as the early diagnosis of endocrine disorders and timely treatment of these complications can improve overall states of health and the quality of life of individuals belonging to this vulnerable population.

## Conflict of Interest Statement

Dr. Wassim Chemaitilly has accepted speaker fees from Novo Nordisk and JCR Pharmaceuticals (Japan). Dr. Nicole Barnes has no conflicts of interest to disclose.
